# Factors Statistically Predicting At-Risk/Problematic Internet Use in a Sample of Young Adolescent Boys and Girls in South Korea

**DOI:** 10.3389/fpsyt.2018.00351

**Published:** 2018-08-07

**Authors:** Yoon-Jung Kim, Daeyoung Roh, Sang-Kyu Lee, Fatih Canan, Marc N. Potenza

**Affiliations:** ^1^Department of Psychiatry, Chuncheon National Hospital, Chuncheon, South Korea; ^2^Mind-Neuromodulation Laboratory, Department of Psychiatry, Hallym University Medical Center, Chuncheon, South Korea; ^3^Department of Psychiatry, Akdeniz University, Antalya, Turkey; ^4^Department of Psychiatry, Yale School of Medicine, Yale University, New Haven, CT, United States; ^5^Neuroscience and Child Study, Yale School of Medicine, Yale University, New Haven, CT, United States; ^6^National Center on Addiction and Substance Abuse (CASA Columbia), Yale School of Medicine, Yale University, New Haven, CT, United States; ^7^Connecticut Mental Health Center, New Haven, CT, United States; ^8^Connecticut Council on Problem Gambling, Wethersfield, CT, United States; ^9^The Connecticut Department of Mental Health and Addiction Services, Connecticut Council on Problem Gambling, Wethersfield, CT, United States

**Keywords:** adolescent, gender difference, addictive behavior, biomarkers, exploratory behavior, internet

## Abstract

**Aims:** This study aimed to investigate in a gender-sensitive manner factors related to at-risk/problematic Internet use (ARPIU) in a sample of young Korean adolescents. Given prior findings, we hypothesized we would observe specific temperamental, social and biological measures that would statistically predict ARPIU in boys and girls, respectively.

**Method:** Subjects included 653 middle-school students from Chuncheon, Korea who completed measures assessing Internet addiction, mood, temperament, and social interactions. Finger digit (2D:4D) ratios were also assessed. Chi-square and logistic regression models were conducted.

**Results:** Among boys and girls, the ARPIU and non-ARPIU groups showed differences in temperament, mood, social tendencies, and gaming behaviors. In boys, IAT correlated inversely with the 2D:4D digit ratio and novelty-seeking and positively with reward-dependence scores when controlling for BDI scores; these relationships were not found in girls. Multivariate analyses showed that among boys, novelty-seeking, harm avoidance, self-transcendence, and daily time spent gaming statistically predicted ARPIU. Among girls, daily time spent gaming, number of best friends, self-directedness, and cooperation statistically predicted ARPIU.

**Conclusion:** ARPIU was linked to specific temperamental, behavioral and biological characteristics, with specific relationships observed in boys and girls. Specific risk factors may exist for boys and girls with respect to their propensities to developing ARPIU, suggesting the need for gender-sensitive approaches to prevent ARPIU in youth.

## Introduction

Concerns have been raised that specific types and patterns of Internet use may be problematic for adolescents and their development, and specific youth may be particularly vulnerable to developing problematic Internet use (PIU). As such, identifying features associated with PIU may help in prevention and other intervention efforts. Considering gender-related differences is important in targeting such interventions and optimizing care. In some studies, PIU has been reported to approximately two-fold more frequent in males as compared to females (1, 2), although other studies report less robust gender-related differences in prevalence (3). Boys and girls also differ with respect to types of online activities performed; for example, boys may spend more time gaming and girls may spend more time chatting and blogging (4). The latter findings suggest that PIU in girls may particularly related to social factors, consistent with females as compared with males tending to report more problems with social networking (5). Gender-related differences have been observed in temperamental features linked to PIU. Temperament may involve biological-based individual differences in reactivity and self-regulation (6) and may be assessed using instruments such as Cloninger's temperament character inventory (TCI) (7). Using the TCI, PIU in boys was linked to high novelty-seeking and harm-avoidance and low reward-dependence, and in girls PIU was linked to high harm-avoidance, low reward-dependence and cooperativeness (8).

Other factors may relate importantly to PIU in a gender-specific fashion. One biologically based physical characteristic, the second to fourth digit length ratio (2D:4D ratio), has been proposed as a possible marker linked to psychological characteristics in males. The 2D:4D ratio differs according to gender/sex, with males typically exhibiting lower ratios (9, 10). This sexually dimorphic feature typically appears around the 9-week point of the prenatal period, and it remains relatively constant from week 14 to adulthood (10, 11). The 2D:4D ratio in part reflects the level of exposure to sex hormones during the prenatal period, with high exposures to testosterone or high sensitivities of testosterone receptors prenatally lowering 2D:4D ratios in males and higher estrogen levels in females increasing 2D:4D ratios (11–13). Several previous studies had conducted to investigate the relationship between 2D:4D ratio and PIU. PIU (14) and problematic video-gaming (15) were associated with low 2D:4D ratios. However, Muller et al. (16) reported the association of 2D:4D and Internet gaming disorder only in females.

Given the above findings, additional research into behavioral, psychological, and biologically based physical factors related to PIU in males and females warrant additional investigation. The most appropriate thresholding for PIU has been debated (17), and data suggest that the more stringent thresholding used in diagnostic criteria for behavioral as compared to substance addictions may miss clinically relevant groups (18). Given that youth may have their developmental trajectories may be negatively impacted by engagement in sub-diagnostic engagement in risk behaviors, we opted to investigate PIU using a relatively liberal threshold (which we term at-risk/PIU, or ARPIU), as we have in prior studies (19–22).

In the current study, we aimed to assess a multiple behavioral, temperamental and physical factors may relate to ARPIU in male and female young middle-school students in South Korea. We based our hypotheses on existing data on PIU from different counties and age groups, with the understanding that environmental, cultural and other factors may have important influences that may alter relationships. We hypothesized that we would identify different factors related to ARPIU in boys and girls. For example, we hypothesized that time spent gaming, novelty-seeking, and the 2D:4D ratio would be related to ARPIU in boys and not girls, whereas time spent chatting and other social factors would be related to ARPIU in girls but not boys.

## Methods

### Participants

This study involved 665 junior middle-school students (in second and third grades) who resided in Chuncheon city, Gangwon province, South Korea. Two classrooms by school and grade were randomly selected. Participants voluntarily accepted to take the survey and were informed of its purpose and methods. Data were analyzed from 653 students [male: 388 (59.3%), female: 265 (40.5%)] due to exclusion of left-handed individuals (12 students, 2%) given assessment of 2D:4D ratio on the right hand only. The survey was conducted from April 1st, 2013 to June 30th, 2013. The study was approved by the Institutional Review Board (IRB) of Hallym University Chuncheon Sacred Heart Hospital. Each participant submitted a written informed consent form after receiving a full explanation of the study's purpose and procedure. Before this process, letters were sent through the school to parents informing them of the purpose of the survey, its methodology, and the procedure by which they could deny permission for their child to participate in the survey if they wished their child to be excluded, as has been done previously (3).

### Questionnaire components

Participants were asked to complete questions regarding general demographic characteristics including age, gender, self-rated school achievement (above 10% highest, 11–30%, 31–70%, 71–90%, below 10% lowest), the status of family and friends (“living with parents,” “experience of having been bullied by others or having bullied others,” and “number of best friends”), unhealthy behaviors like drinking and smoking (“experience of drinking,” “experience of smoking”), and online activities (“purpose of using Internet,” “self-assessment of Internet addiction,” “Need to use the Internet,” “average minutes of daily Internet use,” and “average minutes usually spend daily playing games on the Internet”).

### Internet addiction test (IAT)

The Internet addiction test (IAT) consists of 20 questions using Likert responses (23) and was adapted for use in Korean populations (24). The IAT assesses multiple domains related to PIU including obsessive behaviors, financial losses, underachievement, negligence at home, problems with interpersonal relationships, behavioral problems and emotional changes. A score of 50 or more on the IAT was used to define at-risk problematic Internet use (ARPIU), as done previously (23). The internal consistency as measured using Cronbach's for the IAT was 0.92.

### Junior temperament and character inventory (JTCI)

Following the Temperament and Character Inventory (TCI) developed by Cloninger for adults (7), the Junior Temperament Character Inventory (JTCI) was developed for youth (25). This study used a Korean version of the questionnaire (26). The JTCI evaluates personality, divided into four temperaments and three characteristics. Temperaments consist of novelty-seeking, harm-avoidance, reward-dependence, and persistence. Characteristics consist of self-directedness, cooperativeness, and self-transcendence. The JTCI consists of a total of 108 yes/no questions. The internal consistency as measured using Cronbach's for the JTCI was 0.76.

### Depression scale

The Beck Depression Inventory (BDI) as adapted by Lee (27) was used. The instrument consists of questions along a four-point scale, with the minimum total score being zero points and the maximum 63 points. The scale includes 21 questions regarding emotional, cognitive, motivational and physical aspects of depression. Higher scores reflect more severe depression (Cronbach's Alpha coefficient: 0.86).

### Measurement of 2D:4D ratio

As described previously (10), finger length was measured by putting the right hand on the floor with the right palm facing up, spreading the thumb and collecting the remaining four fingers. The second (index) and fourth (ring) finger length was directly measured from the tip of the finger to the basal crease up to 0.01 mm using a Vernier caliper (28). This measure was conducted by two separate investigators and calculated the mean of the two measurements. The correlations between the two raters was 0.93, indicating excellent inter-rater reliability.

### Study procedure

Surveys and tests were conducted in classrooms. To receive sincere answers on the survey, questionnaires were collected without providing any personally identifiable information. Prior to the surveys and tests, the prepared instruction regarding the intent, purpose and the guaranteed anonymity of the survey was given, and the survey was completed over a 50-min period.

### Statistics

Two groups were generated reflecting an ARPIU group and non-ARPIU group. *T*-test and chi-square analyses were performed to investigate between-group differences on demographic factors and patterns of Internet use. Correlation analyses (Pearson's) and partial correlations adjusted for BDI scores were used to investigate relationships between IAT, JTCI, and 2D:4D ratio measures. Multivariate binary logistic regression models were analyzed to identify factors statistically predictive of ARPIU.

## Results

### Sociodemographic characteristics and patterns of internet use by ARPIU overall and by gender

The ARPIU group was comprised 32.6% of the sample and was predominately male (42.3% of boys vs. 18.5% of girls, χ^2^ = 40.5, *p* < 0.01). The ARPIU and non-ARPIU groups showed largely similar sociodemographic characteristics with respect to household structure, academic achievement, drinking and smoking behaviors, and numbers of best friends (Table 1). The ARPIU and non-ARPIU differed on Internet-use-related characteristics, with the ARPIU group being more likely to report gaming, having experience buying gaming items, feeling like they were addicted to the Internet, and having felt a need to cut back on their Internet use (Table 1). These findings largely extended to both boys and girls. However, among girls, those with ARPIU differed from those without ARPIU on numbers of best friends, with the non-ARPIU appearing more likely to report four or more best friends (Table 1).

**Table 1:** Sociodemographic characteristics and internet-use behaviors stratified by ARPIU-involvement status and gender.

**Variable**	**Total [*****N*** **(%)]**		**χ^2^**	***p*-value**	**Boys [*****N*** **(%)]**		**χ^2^**	***p*-value**	**Girls [*****N*** **(%)]**		**χ^2^**	***p*-value**
	**ARPIU 213 (32.6)**	**Non-ARPIU 440 (67.4)**			**ARPIU 164 (42.3)**	**Non-ARPIU 224 (57.7)**			**ARPIU 49 (18.5)**	**Non-ARPIU 216 (81.5)**		
Living with parents (Yes)	188 (87.9)	396 (90.9)	0.937	0.63	149 (90.99)	209 (93.3)	0.80	0.67	39 (79.6)	187 (86.6)	2.48	0.29
Academic average grade												
Above 10%	21 (9.9)	46 (10.5)	7.33	0.12	17 (10.4)	26 (11.6)	6.56	0.16	4 (8.2)	20 (9.3)	5.35	0.25
11–30%	33 (15.5)	96 (21.9)			28 (17.1)	56 (25.0)			5 (10.2)	40 (18.6)		
31–70%	87 (40.8)	181 (41.2)			69 (42.1)	88 (39.3)			18 (36.7)	93 (43.3)		
71–90%	55 (25.8)	97 (22.1)			37 (22.6)	46 (20.5)			18 (36.7)	51 (23.7)		
Below 10%	17 (8.0)	19 (4.3)			13 (7.9)	8 (3.6)			4 (8.2)	11 (5.1)		
Drinking, life time (Yes)	43 (20.1)	86 (19.5)	0.03	0.47	34 (20.7)	58 (25.9)	1.39	0.14	8 (16.3)	28 (13.0)	0.39	0.34
Smoking, life time (Yes)	62 (29.0)	97 (22.1)	3.63	0.06	58 (35.4)	73 (32.7)	0.29	0.33	4 (8.2)	24 (11.2)	1.10	0.22
Number, best friends												
One or less	13 (7.6)	31 (8.1)	0.79	0.85	8 (6.2)	11 (5.7)	0.57	0.90	5 (12.5)	20 (10.5)	10.65	0.01
2–3 persons	75 (43.9)	153 (39.8)			48 (36.9)	77 (39.7)			26 (65.0)	76 (40.0)		
4–5 persons	45 (26.3)	108 (28.1)			38 (29.2)	50 (25.8)			7 (17.5)	58 (30.5)		
More than five persons	38 (22.2)	92 (24.0)			36 (29.7)	56 (28.9)			2 (5.0)	36 (18.9)		
Purpose of using internet												
Searching information	29 (14.3)	124 (32.0)	75.94	<0.01	15 (9.5)	45 (21.2)	36.75	<0.01	14 (31.1)	79 (45.1)	11.69	0.01
Chatting, mailing	15 (7.4)	26 (6.7)			8 (5.1)	12 (5.7)			7 (15.6)	14 (8.0)		
Gaming	137 (67.5)	122 (31.5)			128 (81.0)	111 (52.4)			9 (20.0)	11 (6.3)		
Others	22 (10.8)	115 (29.7)			7 (4.4)	44 (20.8)			15 (33.3)	71 (40.6)		
Experienced buying game-items (Yes)	141 (66.5)	202 (46.0)	24.24	<0.01	116 (71.6)	128 (57.4)	8.16	<0.01	25 (51.0)	74 (34.3)	5.52	0.06
Felt being addicted to internet (Yes)	73 (34.4)	32 (7.3)	77.62	<0.01	59 (36.4)	22 (9.8)	40.11	<0.01	14 (28.6)	10 (4.7)	27.46	<0.01
Need to cut down using internet (Yes)	130 (61.0)	111 (25.3)	78.30	<0.01	97 (59.5)	68 (30.5)	32.40	<0.01	32 (65.3)	43 (20.0)	40.27	<0.01

### Comparisons of internet use, JTCI and 2D:4D between the ARPIU and non-ARPIU groups overall and by gender

The ARPIU group as compared to the non-ARPIU reported spending more time per day using the Internet and gaming, more depression, and greater novelty seeking, harm avoidance and self-transcendence (Table 2). The ARPIU group as compared to the non-ARPIU also reported less reward dependence, persistence, self-directedness and cooperativeness, and demonstrated lower 2D:4D scores (Table 2). While all of these relationships were also found among boys and most were found between girls, the ARPIU and non-ARPIU did not differ at *p* < 0.05 on the measures of novelty seeking, persistence, self-directedness and cooperativeness, and their 2D:4D ratios were not different (Table 2).

**Table 2:** Comparisons of BDI, TCI, and 2D:4D ratio by gender.

	**Total sample (*****N*** = **653)**			**Boys (*****N*** = **388)**			**Girls (*****N*** = **265)**		
	**ARPIU (*N* = 213)**	**Non-ARPIU (*N* = 440)**		**ARPIU (*N* = 164)**	**Non-ARPIU (*N* = 224)**		**ARPIU (*N* = 49)**	**Non-ARPIU (*N* = 216)**	
	**Mean ± SD**	**Mean ± SD**	***p*-value**	**Mean ± SD**	**Mean ± SD**	***p*-value**	**Mean ± SD**	**Mean ± SD**	***p*-value**
Time for internet (minutes)	137.47 ± 96.27	83.96 ± 72.32	<0.01	141.10 ± 101.13	89.28 ± 79.40	<0.01	126.91 ± 78.16	78.31 ± 63.65	< 0.01
Time for gaming (minutes)	93.90 ± 83.21	38.58 ± 55.58	<0.01	107.61 ± 83.09	58.17 ± 63.31	<0.01	48.83 ± 67.46	17.75 ± 35.80	< 0.01
BDI	11.74 ± 6.53	9.20 ± 6.65	<0.01	10.77 ± 5.46	8.57 ± 5.77	<0.01	14.15 ± 8.32	9.74 ± 7.28	<0.01
NS	8.70 ± 3.08	7.65 ± 3.04	<0.01	8.68 ± 3.03	7.48 ± 3.20	<0.01	8.73 ± 3.29	7.84 ± 2.87	0.06
HA	12.37 ± 4.34	10.55 ± 4.49	<0.01	12.24 ± 4.38	9.95 ± 4.53	<0.01	12.84 ± 4.26	11.18 ± 4.38	0.02
RD	4.97 ± 1.88	5.85 ± 1.97	<0.01	4.97 ± 1.88	5.56 ± 1.93	<0.01	4.98 ± 1.90	6.15 ± 1.98	< 0.01
PS	2.84 ± 1.26	3.26 ± 1.37	<0.01	2.92 ± 1.23	3.52 ± 1.35	<0.01	2.59 ± 1.37	2.99 ± 1.33	0.06
SD	9.68 ± 3.66	12.01 ± 3.66	<0.01	10.01 ± 3.63	12.41 ± 3.71	<0.01	8.57 ± 3.57	11.59 ± 3.56	< 0.01
CO	12.50 ± 3.26	13.78 ± 3.03	<0.01	12.22 ± 3.23	13.63 ± 3.14	<0.01	13.41 ± 3.23	13.92 ± 2.90	0.28
ST	4.83 ± 2.35	4.31 ± 2.40	<0.01	4.82 ± 2.34	4.27 ± 2.53	0.03	4.88 ± 2.45	4.34 ± 2.25	0.14
2D:4D	0.96 ± 0.04	0.97 ± 0.04	<0.01	0.949 ± 0.03	0.955 ± 0.03	0.05	0.981 ± 0.04	0.985 ± 0.05	0.58

*Tested by Independent sample t-test. BDI, Beck depression inventory; JTCI, junior temperament character inventory; NS, novelty seeking; HA, harm avoidance; RD, reward dependence; P, persistence; SD, self-directedness; CO, cooperativeness; ST, self-transcendence*.

### Correlations among IAT scores, BDI scores, JTCI scores, and 2D:4D ratios overall and by gender

IAT scores correlated with BDI scores, scores on all subscales of the JTCI, and the 2D:4D digit ratio in the overall sample (Table 3). These findings extended to both boys and girls with the exception of novelty seeking, self-transcendence, and 2D:4D ratio in girls not being significant at *p* < 0.05 (Table 3). The 2D:4D ratio was also modestly correlated with BDI scores and reward dependence, cooperativeness in the overall sample only (Table 3). However, when controlling for BDI scores, the 2D:4D digit ratio remained correlated with IAT and reward dependence scores overall and among boys, and was also correlated with novelty-seeking overall and among boys (Table 3).

**Table 3:** Correlations among IAT scores, BDI scores, JTCI scores, and 2D:4D ratios.

		**IAT**	**BDI**	**NS**	**HA**	**RD**	**P**	**SD**	**CO**	**ST**
BDI	T	0.226^**^								
	B	0.215^**^								
	G	0.310^**^								
NS	T	0.158^**^	0.247^**^							
	B	0.195^**^	0.312^**^							
	G	0.109	0.198^**^							
HA	T	0.222^**^	0.435^**^	0.008						
	B	0.274^**^	0.458^**^	0.052						
	G	0.215^**^	0.423^**^	−0.062						
RD	T	−0.257^**^	−0.337^**^	−0.140^**^	−0.312^**^					
	B	−0.191^**^	−0.322^**^	−0.151^**^	−0.387^**^					
	G	−0.275^**^	−0.379^**^	−0.125^*^	−0.238^**^					
PS	T	−0.184^**^	−0.192^**^	−0.307^**^	−0.175^**^	0.135^**^				
	B	−0.278^**^	−0.247^**^	−0.315^**^	−0.161^**^	0.144^**^				
	G	−0.167^**^	−0.137^*^	−0.303^**^	−0.181^**^	0.179^**^				
SD	T	−0.299^**^	−0.491^**^	−0.341^**^	−0.468^**^	0.295^**^	0.432^**^			
	B	−0.310^**^	−0.487^**^	−0.349^**^	−0.446^**^	0.321^**^	0.403^**^			
	G	−0.357^**^	−0.510^**^	−0.331^**^	−0.499^**^	0.283^**^	0.470^**^			
CO	T	−0.237^**^	−0.272^**^	−0.318^**^	−0.305^**^	0.392^**^	0.217^**^	0.419^**^		
	B	−0.241^**^	−0.326^**^	−0.339^**^	−0.362^**^	0.392^**^	0.204^**^	0.442^**^		
	G	−0.157^**^	−0.251^**^	−0.287^**^	−0.241^**^	0.364^**^	0.288^**^	0.407^**^		
ST	T	0.133^**^	0.167^**^	−0.037	0.074	−0.003	0.011	−0.178^**^	0.023	
	B	0.148^**^	0.146^**^	−0.045	0.015	0.013	0.036	−0.196^**^	0.077	
	G	0.112	0.200^**^	−0.024	0.172^**^	−0.020	−0.026	−0.153^*^	−0.063	
2D:4D	T	−0.156^**^	0.096^*^	−0.074	0.069	0.101^**^	−0.019	−0.044	0.081^*^	0.018
	B	−0.139^**^	0.059	−0.055	−0.015	0.063	0.022	0.027	0.042	−0.033
	G	0.035	0.082	−0.110	0.116	0.031	0.045	−0.087	0.031	0.089
2D:4D^#^	T	−0.189^**^		−0.152^**^	−0.056	0.121^*^	0.048	0.055	0.095	0.061
	B	−0.174^**^		−0.141^*^	−0.028	0.138^*^	0.038	0.055	0.090	0.015
	G	0.010		−0.109	0.102	0.059	0.054	−0.056	0.036	0.074

* p < 0.05,

** *p < 0.01, tested by Pearson's correlation*,

# *Partial correlation between IAT scores, JTCI scores, and 2D:4D ratios when con trolling for BDI scores. T, total sample; B, only boys; G, only girls; IAT, Young's internet addiction test; BDI, Beck depression inventory; JTCI, junior temperament character inventory; NS, novelty seeking; HA, harm avoidance; RD, reward dependence; P, persistence; SD, self-directedness; CO, cooperativeness; ST, self-transcendence*.

### Factors statistically predicting ARPIU by gender

A stepwise multiple regression analysis was performed using the independent variables of Internet using characters, BDI, Temperament and Character factors, Finger digit ratio which showed significant correlation with the online game addiction score from Pearson's correlation analysis by gender. Table 4 shows the results of the multivariate logistic regression analysis of factors statistically predicting ARPIU in boys and girls. The amount of time spent playing games on the Internet was a predicting factor for ARPIU in both gender groups (Table 4). In boys, novelty seeking, harm avoidance and self-transcendence were predictive factors; in girls, self-directedness and the number of best friends were predictive factors (Table 4).

**Table 4:** Factors statistically predicting ARPIU by gender.

**Gender**	**Factors**	***B***	**S.E**.	**Wald**	***p*-value**	**Exp (*B*)**	**95% C.I**.
Boy	Time spent gaming	0.006	0.002	11.449	0.001	1.006	1.003–1.010
	NS	0.136	0.043	9.831	0.002	1.146	1.052–1.247
	HA	0.118	0.031	14.676	<0.001	1.126	1.060–1.196
	ST	0.152	0.057	7.056	0.008	1.164	1.041–1.303
Girl	Time spent gaming	0.009	0.004	4.938	0.026	1.009	1.001–1.017
	Number of best-friends (Ref: less than one persons)	−0.567	0.256	4.915	0.027	0.567	0.343–0.936
	SD	−0.302	0.068	19.639	<0.001	0.739	0.647–0.845

*Multivariate stepwise binary logistic regression analysis. B, β values are the estimated unstandardized regression coefficients. S.E., standard error; C.I., confidence interval; NS, novelty seeking; HA, harm avoidance; RD, reward dependence; SD, self-directedness; CO, Cooperativeness; ST, self-transcendence*.

## Discussion

The present study investigated factors related to ARPIU in a sample of young adolescent Korean students, with a focus on gender. Our *a priori* hypotheses were partially supported in that time spent gaming and novelty seeking were factors predictive of ARPIU in boys and social factors were linked in girls. However, other aspects were not hypothesized, such as that time spent gaming would also predict ARPIU in girls. Implications of the findings are discussed below.

### Purposes for using the internet

This study's results showed that the non-ARPIU group used the Internet mainly for searching for information (32.0%) and gaming (31.5%), whereas the ARPIU group used the Internet mainly for gaming (67.5%), especially in boys (81.0%). In ARPIU girls, the Internet was used mainly for searching for information (31.1%), gaming (only 20.0%), chatting (15.6%), and other purposes (33.3%). These findings are thus largely similar to those reported previously from other samples (29). Gender may relate importantly to the reasons underlying Internet use and the patterns and types of Internet use. One hypothesis was that males would be more likely to use the Internet problematically for seeking new and exciting experiences, for example through gaming, while females would be more likely to use the Internet problematically when connecting socially through chatting and other means. Other findings only partially support our hypotheses. Whereas for boys novelty seeking and time spent gaming were factors linked to ARPIU and statistically predicted ARPIU in logistic regression analyses, chatting was not linked *per se* to ARPIU in girls (in part perhaps due to the nature of the assessment question). However, the finding linking fewer best friends to ARPIU in girls, with this measure statistically predicting ARPIU in girls in the logistic regression models, suggest that social factors may be particularly relevant to ARPIU among young Korean adolescents. Given the increasing Internet use over time, it will be important to monitor for ARPIU in youth (and other age groups), particularly as the majority of the adolescent boys (90.2%) in South Korea play on-line games (30) and that the use of the Internet to connect socially (e.g., via social networking and other processes) has also been increasing over time (31).

### Depression, temperament and character, and digit ratio

Correlations among IAT scores, BDI scores, JTCI scores, and 2D:4D ratios were identified in this study. The IAT scores showed positive correlations with BDI, consistent with prior findings in other populations (32, 33). Perhaps due to depressive symptoms, adolescents might experience difficulties in learning and engaging in social activities, and thus may use the Internet as a means of avoiding stress (34).

Internet addiction scores showed positive correlations with novelty-seeking, harm-avoidance, and self-transcendence scores and negative correlations with reward-dependence, persistence, self-directedness, and cooperativeness scores and 2D:4D ratio in boys; however, in girls, novelty-seeking, self-transcendence, and 2D:4D ratio relationships were not statistically significant at *p* < 0.05. The positive correlation between Internet addiction score and novelty-seeking were in line with prior findings linking sensation-seeking and impulsivity to Internet addiction (32, 33), and the positive correlation between Internet addiction scores and harm-avoidance (35) suggest that individuals experiencing difficulty in making interpersonal relationships with others in real life (perhaps due to shyness, anxiety, and difficulties in social adaptation) may be particularly vulnerable to using the Internet in problematic fashions.

Reward dependence, persistence, self-directedness, cooperativeness had significant correlations with IAT score in both gender groups. Reward dependence reflects a propensity for being cynical, apathetic, insensitive to social situations, and indecisive and not feeling positive emotions easily. These results are consistent with prior findings reporting that Internet addiction is linked to loneliness and limited social relationships (35). Persistence showed negative correlations with Internet addiction scores. This finding suggests that tendencies for Internet addiction may be linked to not feeling a motivation to act when no reward or a small reward is given; speculatively, individuals with low persistence may turn to the Internet (perhaps for gaming) which may provide more instant gratification. Negative correlations were also seen between Internet addiction and self-directness scores. Low self-directness reflects a tendency to pursue shorter- as opposed to than longer-term goals; as such individuals with ARPIU may seek goals through immediate responses from the Internet (36). A negative correlation was seen between Internet addiction and cooperativeness. Low cooperativeness may interfere with the establishment of social relationships, and such tendencies may lead to social isolation and tendencies to use the Internet (over direct interpersonal social relations) and develop ARPIU. Given the similarities in these relationships across gender groups, these features may reflect general tendencies linked to ARPIU.

A negative correlation was seen between Internet addiction scores and 2D:4D ratio in the overall sample and in boys but not girls. Further, the finding persisted when controlling for severity of depressive features. These results resonate with findings linking low 2D:4D ratios to sensation-seeking and risk-taking (37–39), substance addictions like alcohol dependence (40) and Internet addiction in other groups such as Turkish college students (41). Taken together, multiple factors (male sex, low 2D:4D ratios, and specific temperamental features) may relate importantly to ARPIU.

### Subtyping of internet addiction

The results of this study lay the groundwork for examining the extent to which there may exist subtypes of Internet addiction, similar to those reported in alcohol-use disorders (42). First, factors relating to Internet addiction tendency may vary by gender, with factors like novelty seeking being particularly relevant for males. Second, the biologically influenced 2D:4D ratio, linked to prenatal testosterone exposure, suggests a biological factor linked to novelty seeking and ARPIU in males, particularly when accounting for depressive symptoms. The notion that individuals with high and low depressive features is suggested and warrants additional investigation.

### Limitations

There are limitations to this study. First, the participants included solely junior middle-school students in South Korea. While age may not substantially influence some factors (e.g., 2D:4D ratios), future studies should examine the extent to which the findings extend to other populations (e.g., different ages and cultural backgrounds). Second, only right-handed 2D:4D ratios were assessed. Given potential differences in correlations between psychological measures and handedness (43), future studies should consider handedness. Third, clinical assessment through structured interview was not conducted given subject burden, and future studies should consider more precise psychiatric assessments.

## Conclusion

This study found both gender-specific and shared features across gender groups that related to ARPIU in young adolescents from South Korea. A common finding that time spent gaming on the Internet statistically predicts ARPIU across genders suggest the relevance of public health guidelines related to Internet gaming among youth. The extent to which there may exist subtypes of individuals with Internet-use problems warrants additional study, particularly if such groups may benefit from specific interventions.

## Author contributions

S-KL and MP designed the study. Y-JK wrote the protocol. S-KL, DR, and MP managed the literature searches and analyses (including the statistical analysis). Y-JK, S-KL, and MP wrote the first draft of the manuscript. S-KL and Y-JK managed the entire surveys. S-KL, MP, and FC contributed to the analysis and interpretation of data for the work, revised the work. All authors contributed to and have approved the final manuscript.

### Conflict of interest statement

The authors declare that the research was conducted in the absence of any commercial or financial relationships that could be construed as a potential conflict of interest.

## Abbreviations

BDI: Beck depression inventory
IAT: Young's internet addiction test
TCI: temperament character inventory
JTCI: junior temperament character inventory
NS: novelty seeking
HA: harm avoidance
RD: reward dependence
P: persistence
SD: self-directedness
CO: cooperativeness
ST: self-transcendence
2D: second digit length
4D: fourth digit length.
